# Implementing Fuzzy AHP and FUCOM to evaluate critical success factors for sustained academic quality assurance and ABET accreditation

**DOI:** 10.1371/journal.pone.0239140

**Published:** 2020-09-17

**Authors:** Naim Ahmad, Ayman Qahmash

**Affiliations:** Department of Information Systems, King Khalid University, Abha, Saudi Arabia; University of Defence, SERBIA

## Abstract

The Accreditation Board for Engineering and Technology (ABET) accredits the tertiary education programs in the areas of applied and natural science, computing, engineering, and engineering technology. ABET offers accreditation in the United States and other regions in the world that lack such entities such as Gulf Cooperative Counties (GCC). Though ABET accreditation is voluntary, graduates of the ABET-accredited programs are considered equivalent in knowledge, behaviors, and attitude with global standards. The process of ABET accreditation takes months or years depending upon the gap with readiness and resources. The objective of this study is to compile and prioritize the list of critical success factors (CSFs) to commit resources optimally for sustained academic quality assurance and ABET accreditation. The triangulation research designed has been employed. Firstly, the observation of the ABET accreditation process of multiple programs at King Khalid University (KKU) helped in identifying 11 CSFs in three categories namely Program design and execution, Quality culture and excellence, and Institutional infrastructure and support. Further, these CSFs have been explored in the literature in the area of ABET accreditation. Finally, the research employs a fuzzy analytical hierarchy process (Fuzzy AHP) and full consistency method (FUCOM) to rank the relative importance of these CSFs and their dimensions for sustained academic quality assurance and ABET accreditation. The incorporation of these CSFs will help institutions in the GCC and other regions to get their academic programs ABET-accredited in an optimal manner.

## Introduction

Historically, engineering education has gone through the phases of evolution, regulation, and quality control through accreditation [1]. Accreditation of engineering and computing education through some global benchmarks has become essential to ensure equivalence and quality in the programs of study. The universities in the Gulf Cooperative Council (GCC) region are increasingly getting their engineering and computing programs accredited by Accreditation Board for Engineering and Technology (ABET) in addition to their respective national accreditation agencies. ABET is a founding signatory organization of United States for the Washington Accord, a constituent of International Engineering Alliance (IEA). ABET accredits college and university programs in the disciplines of applied and natural science, computing, engineering and engineering technology at the associate, bachelor’s and master’s degree levels. As per current figures at ABET website [2] in the GCC region, 25 Saudi Arabian, 15 UAE, 5 Kuwaiti, 2 Qatari, 2 Bahraini and 1 Omani universities have got their programs ABET-accredited. Although, in Saudi Arabia there is a government body National Center for Academic Accreditation and Assessment (NCAAA) under Education Evaluation Commission (EEC) in the Ministry of Education (MoE) that accredits programs and institutions. NCAAA is not yet a signatory of Washington Accord hence, MoE encourages institutions to get their programs ABET-accredited to achieve international equivalence. The article [3] describes the level of similarity between NCAAA and ABET.

ABET focuses on outcome-based learning (OBL), a marked shift in engineering education from content-based learning (CBL) in the late 1990s and early 2000s [4, 5]. In other words evaluation of what has been learned rather than on what has been taught [6]. It encourages excellence in technical education through Continuous quality improvement (CQI) processes [7]. The objectives of ABET accreditation includes achievements of program objectives, graduates readiness for professional practice, improvement of education and adoption of innovative approaches in education [8, 9]. It provides a self-study questionnaire template to guide the applying institution to develop its self-study report (SSR). It consists of eight main criteria student, program educational objectives, student outcomes, continuous improvement, curriculum, faculty, facilities and institutional support. Additionally, it requires to provide background information and several documents in the attachments. Prior to on-site visit by ABET team, institution is supposed to first submit readiness review report, very similar to self-study report. This is to avoid unnecessary wastage of resources and time. The process of ABET accreditation also involves the peer review by the visiting team of academicians and professionals. On successful completion of all the formalities program is ABET-accredited and next general review is scheduled during a period of six years.

The ABET accreditation process is generally found to be cumbersome, resource draining and time consuming. Mostly previous studies in the area of ABET have focused on student learning outcome, course design, assessments and CQI in different disciplines of engineering. And some studies have also discussed the issues of program objectives, review from stakeholders, and teaching strategies and research. There are also studies that have surveyed the existing program curriculums and identified the lack of importance to the areas of professional skills development, and systems and sustainability education [10]. The success in ABET accreditation has also been attributed to emphasis on hands-on practical education, well-involved relation with local industry, well-planned ABET-related assessment procedures and surveys [11]. This research attempts to present holistic approach to establish the academic quality assurance and achieve ABET accreditation. ABET accreditation may be considered as bringing in global quality standards in the academic programs. Therefore, the process needs to be embedded in a smooth and seamless fashion leaving the desired permanent change rather than temporal disruption. Further the academic quality assurance initiative must be integrated and sustained at all times.

The ABET Accreditation process has been very methodical and disciplined at King Khalid University (KKU). It has helped to benchmark different engineering and computing programs against the global standards and improve the shortcomings therein. The objective of this study is to compile the critical success factors (CSFs) essential to achieve and sustain the academic quality assurance and ABET accreditation in a systematic manner. Further, the research also attempts to identify relative importance of CSFs using fuzzy analytical hierarchy process (Fuzzy AHP) and full consistency method (FUCOM) with the help of decision makers. The study also serves the purpose of educating the faculty staff on the relevant critical issues and required modernization in instruction and assessment to make the graduates ready to face the real challenges of workplaces. The outcome of this research will help institutions to manage and commit their resources effectively to achieve and sustain academic quality assurance and ABET accreditation. The rest of the article is organized into following sections: background of ABET, critical success factors for academic quality assurance and ABET accreditation, Program design and execution, Quality culture and excellence, Institutional infrastructure and support, multi-criteria decision making models: Fuzzy AHP and FUCOM, application of Fuzzy AHP and FUCOM, and discussion and conclusions.

## Background of ABET

Earlier in Europe there were mixed approaches in the engineering education such as formal education based on mathematics and science developed in France, apprenticeship-based system developed in England, and their hybrid system [1]. Similarly during 1930s engineering education in United States of America (USA) was more practice oriented and curriculum lacked the scientific and mathematical rigor [4]. Grinter report prepared by a committee appointed by American Society for Engineering Education (ASEE) in 1952, brought sciences, engineering sciences and mathematics into the curriculum. This made United States (US) engineering education similar to that of European in content. On the other hand, the quality of engineering education was being governed through accreditation agencies. Engineers’ Council for Professional Development (ECPD) was founded in 1932 by seven US engineering societies and later in 1980, it changed its name to ABET [2]. In 2001, with the help of Computing Sciences Accreditation Board, ABET formed Computing Accreditation Commission to accredit computing programs [6]. As of now it is a representative of federation of 35 member societies. It accredits academic programs in science, technology, engineering and mathematics (STEM) through four commissions namely applied and natural science, computing, engineering, and engineering technology. As per the latest records till date, it has accredited 4,005 programs at 793 colleges and universities in 32 countries [2]. To achieve this, ABET relies upon a strong team of 2200 volunteers from academia, government and industry known as program evaluators.

ABET had initially in 1930s simple guidelines for engineering programs evaluation and by 1990s basis changed to lengthy prescriptive criteria [6, 12]. That was becoming increasingly counterproductive to address future challenges of engineering education. ABET had to bring in reformations to sustain quality and innovation in engineering programs. In 1994, ABET with National Science Foundation and industry conducted series of workshops and developed engineering criteria 2000 (EC2000). EC2000 parted ways from being very prescriptive and subject matter specified to outcome-based learning and Continuous quality improvement. Further, it mandated to define program objectives to capture unique characteristics and contextual environment. EC2000 identified 11 student learning outcomes that a graduate must exhibit through knowledge, behavior and attitude [13]. Still some engineering departments such as Caltech and Stanford ‘s chemical, are finding it difficult to offer ABET accreditable innovative programs [14]. Therefore, planned to offer non-ABET-accredited programs from 2017. These trends are further making ABET to reduce the prescriptiveness and can be witnessed through the reduction of student outcomes to 7 in engineering and 6 in computing programs from the year 2019–20 [2].

ABET has played significant role in ensuring the quality of engineering programs in the international arena. It had first signed the accord with Canadian Engineering Accreditation Board (CEAB) for mutual recognition agreement (MRA) in the year 1979 [6]. Later six nations United States, Canada, United Kingdom, Ireland, New Zealand and Australia known as founding signatory, signed the Washington Accord in the year 1989. Earlier ABET used to provide substantial equivalency status to the reviewed programs outside USA. Substantial equivalency meant “program is comparable in program content and educational experience, but may differ in format or method of delivery” [2]. Due to ambiguity and non-binding acceptance of substantially equivalent programs [15], it now confers accreditation status to the programs outside USA such as GCC or Latin America [16]. At the first level outside USA, it supports the national accreditation agencies through memorandum of understanding (MOU) to develop the accreditation system, as is the case with NCAAA. It is then up to the agency to apply for MRA to get into multilateral Washington Accord. Currently Washington Accord has 20 signatories and 8 provisional signatories for MRA.

## Methodology

This study follows the triangulation research design to afford a multiple lines of sight or actions on to the problem being studied [17]. Firstly, a qualitative case study approach that tries to list the CSFs observed in the successful ABET accreditation of multiple programs at King Khalid University. Secondly, literature review that explores the identified CSFs in the literature in the field of ABET accreditation. Finally, the application of fuzzy analytical hierarchy process (Fuzzy AHP) and full consistency method (FUCOM) to rank the relative importance of the CSFs. Fuzzy AHP and FUCOM are multi-criteria decision making techniques and explained in the relevant section. Three key personal involved with ABET accreditation process at KKU served as the decision makers to assess the dimensions and CSFs. This study addresses following research questions:

- What are the CSFs for the sustained academic quality assurance and ABET accreditation?
- What is the relative ranking of CSFs for sustained academic quality assurance and ABET accreditation?

Following sub-sections mention different data sources used in this study.

### Case organization

King Khalid University (KKU) is a public university in the Kingdom of Saudi Arabia, established in 1998 by the merger of two older universities. It has set its ambitious vision to be among top 200 global universities by 2030. As of 2019 KKU has nearly 50 colleges, 72,000 plus students and around 7,000 faculty and staff [18]. Till date 9 of the engineering and computing bachelor’s programs at KKU are ABET-accredited.

### Articles from literature

The articles were searched on web of science database with the keyword “Accreditation Board for Engineering and Technology”. In order to keep sample manageable in the review process articles based on significance and recency meaning more than 10 citations or published in the year 2016 or later were selected. The full-length accessible articles were kept on augmented until 7/7/2020 and updated list contained 72 articles and thereof 70 articles were found to be relevant. All 70 articles provided appropriate evidences to support the identified CSFs as discussed in the corresponding sections.

### Experts involved as key people in quality at KKU

Three key people involved in the Quality and ABET Accreditation process at the senior position agreed to serve as decision makers (DMs) to share their expertise. All the DMs were heading the programs’ quality teams, Table 1. Additionally, they were also working at Quality Vice-Presidency and Quality Deanship at KKU. Their expert opinions were sought in the process of pair-wise comparisons of dimensions of CSFs and their CSFs, as illustrated in application of Fuzzy AHP and FUCOM section.

**Table 1:** Profile of decision makers.

Decision Makers	Job Designation	Qualification	Experience in Years (Total/Quality)
DM_1_	Associate Professor	Ph.D.	28/15
DM_2_	Assistant Professor	Ph.D.	16/10
DM_3_	Assistant Professor	Ph.D.	13/8

### Critical success factors for academic quality assurance and ABET accreditation

Accreditation in general may be defined as “a process, based on professional judgment, for evaluating whether or not an educational institution or program meets specified standards of educational quality” [1]. ABET that focuses only on programs defines accreditation as “a review process to determine if educational programs meet defined standards of quality” [2]. In essence ABET is reviewing the qualitative objectives defined for the academic programs and standard student outcomes, and their assessment and evaluation processes in a Continuous quality improvement fashion. Additionally, it also looks into other resources such as finances, facilities, faculty and staff. The preparation to apply for accreditation may take several months to years depending on readiness gap. The whole endeavor requires to be carefully managed to optimize time and resources. A simplified antecedent or significant factor approach such as critical success factor may be adopted.

Critical success factors (CSFs) are defined as ‘‘the limited number of areas in which results, if they are satisfactory, will ensure successful competitive performance for the organization” [19]. CSFs approach has been widely used in the range of disciplines for the adoption of innovation, quality and sustainability initiatives. The literature on ABET shows that the studies that systematically present holistic and generic list of CSFs to acquire ABET accreditation are scant. There is a study [20] that has identified the list of CSFs in this context but focus is on control engineering program success rather than the process of ABET accreditation in general. The observation on the ABET accreditation process in multiple programs for the last five years have led to the identification of following 11 CSFs grouped into three categories. These CSFs have been illustrated with the case organization KKU and literature in the subsequent sections.

- Program design and execution
  ◦ Student management
  ◦ Program vision, mission and objectives
  ◦ Student learning management
  ◦ Curriculum design
  ◦ Continuous quality improvement
- Quality culture and excellence
  ◦ Quality steering team and leader
  ◦ Document orientation and knowledge sharing culture
  ◦ Academic and research excellence
- Institutional infrastructure and support
  ◦ Top management support
  ◦ Institutional quality compliance
  ◦ State of the art facilities

## Program design and execution

This area is most studied in the literature, as it represents the core of program offering and execution. There are five CSFs in this category as explained in the following paragraphs: Student management, Program vision, mission and objectives, Student learning management, Curriculum design, and Continuous quality improvement. These factors discuss the opportunities provided to the students in the program to gain knowledge, skills and attitudes necessary for the professional practice. The observance of these factors at the significant levels is mandatory to achieve ABET accreditation.

### Student management

Student management deals with devising rules and regulations of admission, progression and graduation and their efficient implementation. The most important aspect of admission is the quality criteria for prospective students and process of admission. Similarly, post-admission guidance such as advising, to effectively progress the program based on prerequisite structure and students’ interests and potentials, needs to be well evidenced. Criteria for lateral admissions such as transfer cases must also be specified clearly. Finally, students must also be well informed about the program graduation requirements. This information may also be provided in the form of rule book for the students as in the case of KKU. The sample literature doesn’t mention much of this factor, nonetheless it is first criterion in SSR. Moreover, important decisions with respect to students’ intake and intake quality, regulations for progression and graduation, advising culture and support, transferability rules, incorporation of work experience reflects the good starting point of the program. KKU has a well-structured decision making framework through committees at different levels such as department, college, deanships and vice-presidencies. There are no formal credits for experiential education (co-ops or internships) in engineering and computing programs at KKU but students may earn credit through different proficiency exams. This is somewhat consistent with results of the survey published in 2005, for 90 ABET-accredited civil engineering programs in USA, only 4 had the credit-based co-op requirements [10].

### Program vision, mission and objectives

Academic institutions are setup in line with the national planning agenda for the growth and development of the country. They nurture and supply the required manpower to the industry and society in order to take country forward in the global arena. Same concerns were addressed by the ABET in 1990s by changing the accreditation philosophy from counting of credit hours and detailed specification to assessment of achievement of educational objectives defined in tandem with faculty and industry partners [12]. Therefore, institution’s vision and mission must be reflected in the program educational objectives (PEOs). KKU strives to fulfil regional manpower requirements and fully imbibes the country’s latest vision such as Saudi Vision 2030. PEOs at KKU are developed through synchronisation of missions and goals of university, college and department. Thereafter, these PEOs become the basic guide in the design of curriculum and course learning outcomes (CLOs) and in turn ascertain the desired student learning outcomes or simply student outcomes (SOs). As the SOs are performance-based therefore require more of performance-based assessments such as portfolio, teamwork, oral and written communications, and reflections on real-world experiences for program assessments [8].

### Student learning management

As the education system has changed from the CBL to OBL. It has become imperative that Student learning management must be guided through SOs. It will ensure the necessary knowledge, skills and attitudes in the graduates to start professional practice. SOs may be defined as “observable and measurable manifestation of applied knowledge” [13]. General SOs for different disciplines are provided by ABET [2]. Most important aspect for engineers as opposed to other professions today is to have design skills as reflected in SOs. Some academicians equate design skills to creative thinking and define it to be function of knowledge, imagination and evaluation [21]. Some of the SOs such as involving multidisciplinary teamwork and lifelong learning ensure the generic skills, learning how to learn [22]. Whereas, some of the SOs also aim at development of professional skills such as ethical consideration, problem analysis, impact analysis, multidisciplinary team player, cross-cultural communication, continued professional development, leadership and public service [23, 24]. The leadership skills intertwined in SOs such as initiative/confidence, communication, interpersonal interaction, teamwork and engagement, are highly sought after in entry-level engineering graduates [25]. Similarly, systems thinking skills originated from constructivist theory are also essential as they are highly desirable in complex systems development [26]. The focus of communication skills should not be limited to grammar or format only rather on critical thinking and audience analysis [27].

Ten generic professional profiles or behaviours expected of engineers namely analyst, problem-solver, designer, researcher, communicator, collaborator, leader, self-grower, achiever, practitioner may also help in encapsulating SOs [28]. It is up to the institution to specify SOs in the context of institution and PEOs. Great care must be taken to inculcate creativity and innovation, and personal and interpersonal skills as some studies report the deficiency of them [29]. There are several studies that demonstrate PEO, SO, CLO, KPI relationships [30–32] and automated tools for mappings [33]. At KKU these ABET defined SOs are adapted for each program through departmental quality committees in consultation with faculty. Further key performance indicators (KPIs) are defined against each SO. To measure these KPIs, rubrics as mentioned in the literature [34], are designed.

### Curriculum design

In current educational environment of OBL, Curriculum design has become very systematic. It must incorporate the stipulated components from the program’s disciplines and their underlying mathematics and scientific knowledge. It must produce a study plan consisting of hierarchically structured courses with well-defined course learning outcomes showing correspondence with the SOs. Similarly, a curriculum map may be generated defining places for different learnings and associated activities [27]. The operationalization of OBL requires active involvement of all stakeholders such as students, faculty, educational environment, curriculum and assessment committees, and advisory board [5]. Backward design model is the most suited approach for designing OBL curriculums [35]. Backward design process involves three stages namely identify desired results, determine acceptable evidences (assessments), and plan learning experiences and instructions [36]. Industrial engineering department at Kuwait University used interpretive structural modelling to prioritize SOs during study plan development [37].

Curriculum design is always faced with constraints of resources such as total credits, and rationed allocation of different knowledge domains and disciplines. And there is always pressure to incorporate newer disciplines in the study plan to meet the challenges of work places such as process safety in chemical engineering [38]. Therefore relatively recent theories can be incorporated in the curriculum either in module-based approach (integrating in multiple courses in an interdisciplinary way), stand-alone approach or senior design (capstone project) [34, 39]. Prior to senior year, projects may also be added to link the knowledge with practice [40] through structured and guided design experiences [41, 42]. Pedagogy for the project-based courses may adopt multi-case approach to cover the subjects’ breadth and depth by collaboration on all cases and deep learning on individual cases [43]. For the interdisciplinary fields a live creative environment should be simulated by the close cooperation of multiple departments [44, 45]. For the professional skills, extracurricular courses may be designed using multiple instructional formats such as workshops, presentations, experiential learning from the industry leaders, among others [24]. Education of ethics is also important to expose students with legal, risks, sustainability and integrity issues while designing their solutions [46]. Similarly, entrepreneurship oriented courses can provide unique pedagogical opportunities to create an academic and research environment that will boost innovation, creativity and leadership skills [47, 48] identified integrated engineering public speaking courses boost attitude towards communication that in turn improves sense of engineering identity.

All efforts must be made to take the CLOs at the higher levels of Bloom’s taxonomy (knowledge, comprehension, application, analysis, synthesis and evaluation being the highest) [49] through incorporation of innovative learning strategies and critical reflection [50]. NCAAA has defined national qualification framework (NFQ) for higher education that specifies four learning domains namely knowledge, cognitive skills, interpersonal skills and responsibility, and communication, information technology and numerical skills in the context of engineering and computing programs [51]. At KKU the course specification document defines all the details of the course including definition of CLOs with the keywords showing levels of Bloom’s taxonomy in different learning domains. Further these CLOs are supposed to be mapped to the SOs. ABET accreditation process provided opportunity at KKU to revamp the curriculum to include standard components from different disciplines. The departmental Curriculum and Study Plan committees propose required updates in the curriculum that are reviewed for approval in departmental council meeting.

### Continuous quality improvement

Continuous quality improvement (CQI), requires identification of important stakeholders and program constituencies. Their structured involvement through assessments, reviews and feedback must be sought to make program demand-oriented. Their input may be incorporated in decision making of significant elements of the program such as PEOs, KPIs, SOs, Curriculum, CLOs and program performance. CQI may only be achieved by periodic updates of these elements through direct (students’ performance) and indirect (stakeholders’ feedback) assessments. For instance, at the Department of Engineering Technology, Texas Tech University, 12 different methods of assessments are used in program assessment portfolio: “alumni survey, capstone project report, employer surveys, fundamental review exam, graduate questionnaire, internship report, competitions performance, focus group exit interview, organization participation, seminar attendance, computer skills self-evaluation, pre/post course assessment” [52]. Similarly, Imam Abdulrahman Bin Faisal University, mentions “summative data analysis, formative data analysis, exit exam, faculty survey, and alumni survey” as assessment approaches [53]. The study also identified 7 challenges for designing assessments namely “exhaustive vs. lightweight, top down or bottom up, fair/unbiased, involves faculty members, requires management support, easy to verify, and supportive of continuous improvement activities”. Some studies also mention portfolio as an effective evidence for SOs [8, 27]. Article [54, 55], presents Computing Professional Skills Assessment (CSPA) tool, developed at Zyed University, to assess professional skills. Further, the CQI process must follow close loop concept by developing the action plans to ensure that the desired changes are incorporated at all levels. For CQI, curriculum and instruction, faculty culture, and administrative policies and practices must constantly be tuned to achieve desired SOs [1]. These feedback mechanism though time consuming, serves as the primary actuator for the CQI and must be well demonstrated [56].

One study mentions the details of department of civil and environmental engineering in Seattle University [57]. There are internal constituencies: faculty, academic staff, project centre personnel and students and, external constituencies: project sponsors and liaisons, department advisory board, and the project centre advisory board. The surveys to assess PEOs are sought from alumni every two years, industry funded capstone projects outcomes are assessed by different constituencies throughout the year including sponsors surveys and faculty reflections. At KKU PEOs are generally revised every three years, and other elements yearly or biyearly. And the program constituencies and stakeholders include students, faculty, alumni, employers and external advisory board. The students’ assessments and faculty’s course reports form the basis for the assessments of the CLOs. As per CLO-SO mapping, KPIs of SOs, are measured from the selected courses through rubrics that illustrate upon SOs performance and prospects for improvements. Further, the SO-PEO mapping along with assessments from other constituencies helps in evaluating program performance and need for PEO updates if any.

## Quality culture and excellence

These factors are not much discussed in the literature but play a very important role in sustaining the quality assurance efforts and ABET accreditation. Attaining these factors may pave the road to academic excellence and leadership. There are three factors in this category: Quality steering team and leader, Document orientation and knowledge sharing culture, and Academic and research excellence. Permanent quality structures, information sharing, innovative pedagogies and research orientation will create a very efficient environment to motivate students for effective learning.

### Quality steering team and leader

Every program must have a quality steering team responsible for ensuring the sustainable quality adoption as witnessed in the literature [32]. Constituting the team brings in common minded people together and makes it easier to distribute incentives. Moreover, the team leader may be chosen based on academic, research and experience credentials. Additionally, interpersonal and communication skills are also important. Similarly, leader must be willing to delve into quality matters and help in developing useful templates that may simplify the work for others [32]. The observation at KKU demonstrates that leaders that had tendency to simplify work through templates led to smooth transitions versus those of utilizing delegation authority. Further as per the present norms at KKU, working in quality teams relieves the person from 1 credit-hour to half of academic load based on the faculty’s academic rank and role in the team. The team was actively engaged in motivating and training teaching staff to develop course specification, syllabus, CLOs, CLO-SO mapping, pedagogy and assessments, and course reports that contains section wise data on the execution of course such as results analysis, course delivery, strengths and weaknesses, actions implemented and proposed. The issues of program constituency involvement, program educational objectives review and representative Curriculum design were handled efficiently. This team is also tasked for managing the on-site visit for ABET and NCAAA delegates in addition to the preparation of SSR.

### Document orientation and knowledge sharing culture

The motivation and objectives for documentation should be to facilitate sufficient common understanding among different stakeholders and agencies. Documentation, otherwise can easily exceed the limits and inundate the organization, as pointed in previous reports [1]. One study [32] identified accreditation knowledge dissemination to be an important factor for successful ABET accreditation. [58] presents an intelligent web-based accreditation system to manage the vast amount of data gathering and sharing processes. Academic institutions being part of knowledge industry, have good document orientation. When KKU started its full swing journey in 2013–14 for ABET accreditation, it had necessary documents in place. But during internal review, it was found that some documents had inconsistent and repeated information. This provided an opportunity to inculcate the true spirit of documentation. Easy-access document repository must also be maintained through some opensource platforms such as Google Drive [59]. As in one program quality team is maintaining all the documents in the shared google drive. This provides great motivation to the faculty to contribute quality documents as they can access the complete program information as when needed. Good documentation practices will help in meeting the quality assurance in the long run and avoid the resource drain in reinventing the wheel.

### Academic and research excellence

The traditional lecture-based approach leads to problems of knowledge islands and their integrated relevance to the profession. Academic excellence is the most fundamental aspect of the program. It requires efficient synergies between program design and delivery in order to meet OBL. There are numerous effective learning strategies that have originated in behavioural, cognitive and social psychologies, and education and learning sciences. In the context of engineering and computing, literature mentions following approaches active learning [60], cooperative and collaborative leaning [4], service learning [61] and skills-based learning [62], among others. Active learning methodologies such as problem-based learning [63], project-based learning [64] and case-based learning [46] provide students opportunity to dynamically participate in the learning process as opposed to being passive listeners. One study points that active learning scaffolded through case studies may solve the problems of relevance and disconnected knowledge [60]. Design-based learning, akin to project-based learning, utilizes design process for inquiry, learning and cognition [65]. Some universities offer project-based learning opportunities to inculcate professional skills through industry partners funded by different initiatives such as work integrated learning, innovation and technology assistance program, and talent mobility program [66], and similar other initiatives [67].

Similarly, cooperative and collaborative learning facilitates team-based learning [68] essential to capitalize on collective knowledge and skills. [69] identified the importance of peer-feedback and peer-evaluation to improve team performance. And, service learning [70] offers an exceptional prospect to integrate learning with the societal needs. Further service learning may be blended with other approaches such as project-based or design-based to develop innovation aptitude [65]. Service-learning requires sufficient preparation, service performance and analysis of learning through discussion and reflection [71]. Similarly, project-enhanced combination of project-based and traditional instructional, approach may be utilized in course to impart critical thinking, communication, problem solving and team skills in addition to technical content transfer [40]. Likewise, skills-based learning may be adopted where skills are well defined priori thereafter the associated knowledge is learned. Game-based learning may also be adopted to provide fast and hyper interactive environment [72].

Mini-projects may also provide advantage of breadth, uniformity and accuracy in its assessment over capstone project due to single faculty [56]. And, for the mini-projects or laboratory reports structuring guidelines and emphasis to use appropriate language may improve communication skills and soft skills [73]. Some academicians have proposed using 4H approach that combines the faculties of head, heart, hands and habits to assimilate the knowledge of science and mathematics in the engineering graduates [74]. Write across curriculum and write to learn strategies may also be used to improve cognitive and communication skills through peer review process using tools such as calibrated peer review (CPR) [75]. Value-sensitive design (conceptual, empirical and technical issues investigations) approach may be used to incorporate ethical considerations in the design process [76]. During conceptual investigation 12 human values are considered in design process: human welfare, ownership and property, privacy, freedom from bias, universal usability, trust, autonomy, informed consent, accountability, calmness, identity, and environmental sustainability. For design skills, strategic integrated approach may be adopted to spread it across all years of education by introducing structured, guided and open-ended design experiences [41].

Although there is no research specific criterion for the ABET undergraduate degree programs [15]. Still, to demonstrate the contribution to the discipline and professional development, research becomes an indispensable part. Research keyword is also mentioned in many of the PEOs [5] at various institutions as in KKU also. At times to impart required knowledge and skills in particular domain, only resort available may become funded research projects [77]. KKU also provides funding to the research projects of varied financial budgets ranging from fewer to hundreds of thousands Saudi riyals through the scientific research deanship.

## Institutional infrastructure and support

The high level of institutional maturity will be very conducive for establishing high standards of quality. There are three CFSs in this category: Top management support, Institutional quality compliance, and State of the art facilities. An institution poised for maintaining high quality standards will have positive outlook towards quality, organizational structure to promote quality and develop best resources for the success of the program.

### Top management support

Adoption of innovation, quality and sustainability initiatives requires huge amount of efforts and resources. Endorsements and full-hearted support from top management becomes essential to accommodate required organizational changes and commitment of resources. At KKU highest authority President participates in the quality matters through the Vice Presidency for Development and Quality. This level of involvement is maintained by college dean and department head (program head) by appointing vice-dean quality and program quality coordinator. Similar evidences are available in the literature such as University of South Carolina, College of Engineering appointed Director of Assessment to maintain CQI process [8]. This Vice Presidency manages several deanships, centers and units for academic quality assurance, community services, leadership and entrepreneurship skills development, faculty skills development, strategic planning, corporate governance and international ranking among others. Top management support is also essential to secure necessary financial resources for the professional development of faculty in lieu of quality endeavors.

### Institutional quality compliance

Institutional quality compliance through setting up of a permanent executive division for quality will help in dealing with various agencies such as ABET or NCAAA. The responsibility of this division is to provide a quality related centralised assistance to all the programs offered by different colleges and departments. Northern Border University and King Abdulaziz University mention such unit to manage different ABET requirements [31, 32]. At KKU, there is a full-fledged division known as Academic Development and Quality Deanship (ADQD) under the Vice Presidency of Development and Quality. It creates a conducive environment in colleges and units to adopt quality standards by spreading quality culture and consultancy. Further it oversees quality assurance procedures to help realize their research and educational objectives. It conducts series of workshops on course reports, benchmarking and KPIs, rubrics, CLOs, SOs and assessments. ADQD is also responsible for identifying the necessary resources and their logistics to carry out training. It also, identifies the target programs in the KKU for accreditation and organizes the preliminary activities.

### State of the art facilities

Facilities to carry out program related activities include classrooms, staffrooms, laboratories and library. These facilities must be well furnished and provided with sufficient equipment, efficient computing infrastructure and comprehensive study resources. Modern and updated facilities provide an excellent ambience to carry out instructional and scholarly activities. At KKU due to generous government funding and well-planned development of academic institutions facilities are up to the global standards. E-Learning facilities are managed under the E-Learning Deanship (ELD), established in the year 2005. ELD maintains a robust learning management system (LMS) that offers services for course material sharing, multiple interaction channels, assessments, virtual classroom, classroom capture, learning object repository to share internationally available learning objects. All the courses’ sections are registered each semester in the LMS system and instructors may blend any of the LMS services as suited. KKU has central library, managed under deanship of library affairs, containing hundreds of thousands of books covering all disciplines, digitally manged processes and perfect ambience for acquiring knowledge. Moreover, the Saudi Digital Library (SDL) available to all Saudi universities boast a collection of more than 310,000 e-books, largest in Arab world. SDL has also contracted with more than 300 global publishers such as Elsevier, Springer, Pearson Wiley, Taylor & Francis, Emeralds etc. to offer many other publications such as journals, conference proceedings etc. through its portal. Similarly, General Directorate of Information Technology maintains world class computing, networking and telecommunication resources, and a large collection of e-services in almost every sphere at KKU.

## MCDM models: Fuzzy AHP and FUCOM

The multi-criteria decision making (MCDM) methods help in solving complex problems with conflicting criteria. There are several methods in MCDM that may be applied based on the context of the problem. The analytical hierarchy process (AHP) is one of the subjective MCDM method that has been widely used in resource management problems. The modified Fuzzy AHP method incorporates vagueness in decision making. The Fuzzy AHP has been used due to several advantages such as ease of use, scalability, hierarchical structure adjustable to different size of problems and not data intensive [78]. It has been applied extensively in supplier selection area [79, 80], and in many other areas such as manufacturing, engineering, education, political, social, personal and government [81], and traffic management [82]. In the education field it has been implemented in many areas such as evaluation of teaching performance for teaching quality [83], selection of B-School by an aspirant [84], evaluation of course website quality [85] and E-Learning [86]. There is a relatively new technique known as full consistency method (FUCOM), belonging to same subjective MCDM class, that requires only (*n* − 1) pair-wise comparisons for n criteria against *n* (*n* − 1)/2 comparisons in case of Fuzzy AHP. FUCOM also has built-in consistency and offers the flexibility of measurement scale. Both techniques have been explained in the subsequent sections and their applications is provided in the following section.

### Fuzzy AHP

Fuzzy analytic hierarchy process (Fuzzy AHP) is a fuzzified form of AHP developed by Saaty (1980) [87]. It is a multi-criteria decision making (MCDM) process to prioritize different important attributes in particular area. It uses fuzzy numbers to represent human judgements to compare different attributes. These fuzzy numbers are recorded in fuzzy comparison matrices. Firstly, weights are calculated from fuzzy comparison matrices using one of algorithms such as logarithmic least square, fuzzy extent analysis, fuzzy row sum, fuzzy inverse of column sum, geometric mean and arithmetic mean [88]. This study uses geometric mean approach presented by Buckley (1985) [89] as it outperform other algorithms for smaller size [88]. Following paragraphs briefly explain fuzzy set theory and steps of Buckley’s Fuzzy AHP.

#### Fuzzy set theory

**Fig 1:**
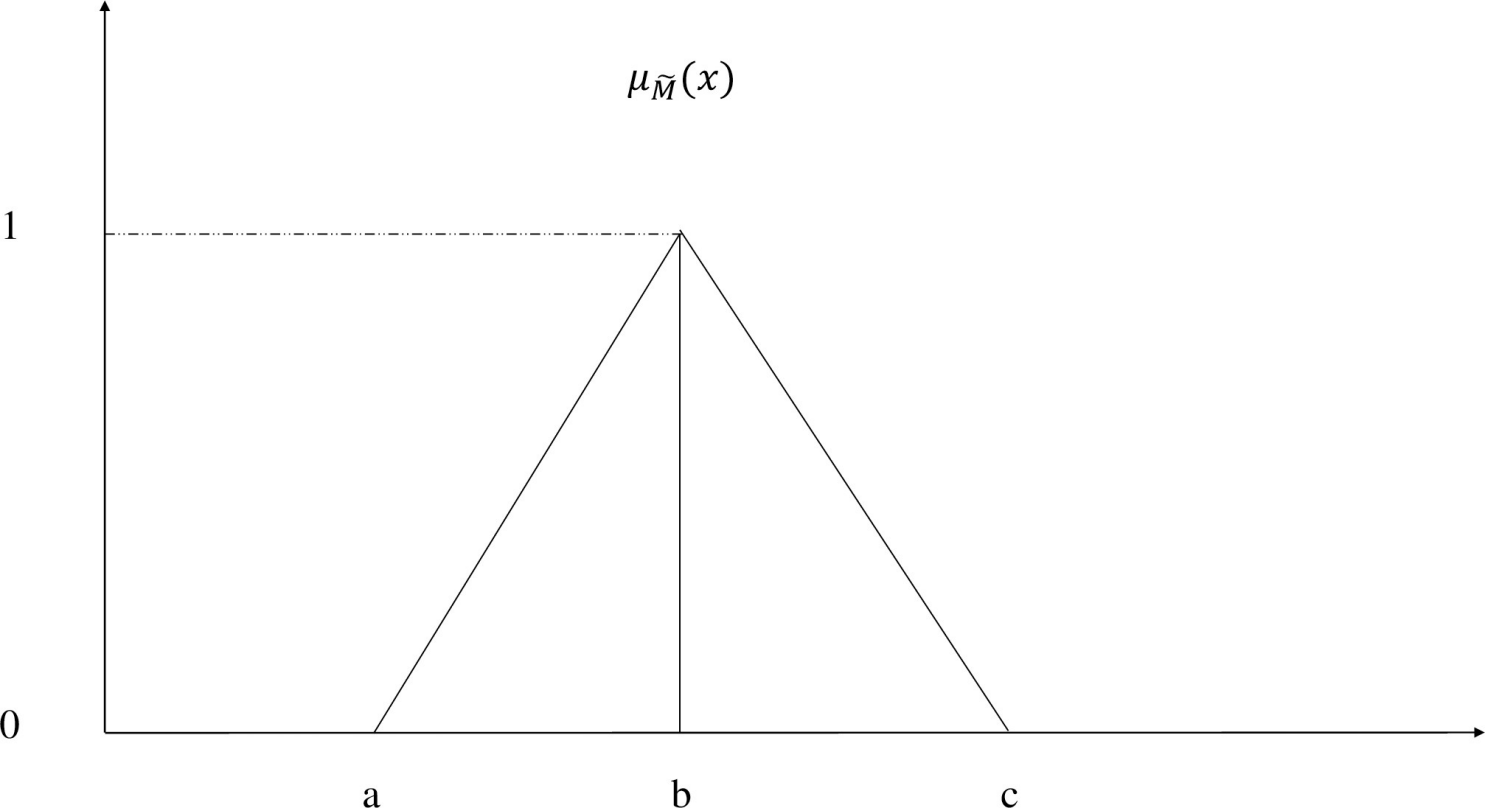
A triangular fuzzy number $\tilde{M}$.

Zadeh (1965) [90] defined fuzzy set theory to represent the vagueness in parameters of decision making. A fuzzy set $\tilde{M}$ is defined by a membership function $\mu_{\tilde{M}}\left(X\right)$ as given in (1). A triangular fuzzy number (TFN) $\tilde{M}$ is defined by a triplet (a, b, c) as shown in Fig 1. And some useful arithmetic operations such as addition, multiplication, scalar multiplication and inverse on TFNs ${\tilde{M}}_{1}=\left(a1,\;b1,\;c1\right)$ and ${\tilde{M}}_{2}=\left(a2,\;b2,\;c2\right)$ are shown in (2) to (5) respectively.

$$\mu_{\tilde{M}}(x)=\{\begin{array}{c} \begin{array}{c} \begin{array}{cc} \frac{x-a}{b-a} & a\leq x\leq b \end{array}\;\\ \begin{array}{cc} \frac{x-c}{b-c} & b\leq x\leq c \end{array} \end{array}\\ \begin{array}{cc} 0 & otherwise \end{array} \end{array}$$ (1)

$${\tilde{M}}_{1}⊕{\tilde{M}}_{2}=(a_{1}+a_{2},b_{1}+b_{2},c_{1}+c_{2})$$ (2)

$${\tilde{M}}_{1}⊗{\tilde{M}}_{2}=(a_{1}a_{2},b_{1}b_{2},c_{1}c_{2})$$ (3)

$$k⊗{\tilde{M}}_{1}=(ka_{1},kb_{1},kc_{1}),\;where\;k>0$$ (4)

$${\tilde{M}}_{1}{}^{-1}=(\frac{1}{c_{1}},\;\frac{1}{b_{1}},\frac{1}{a_{1}})$$ (5)

#### Buckley’s Fuzzy AHP

The five-steps Buckley’s Fuzzy AHP is adapted from [91, 92] as illustrated below. Also, the consistency ratio has been estimated in step 3 as suggested in [93].

**Step 1:** Establishing structure hierarchy for goal. Firstly, the goal of the Fuzzy AHP needs to clearly defined. Thereafter, domains and their factors are listed that contribute in achieving the goal. This requires theoretical base and opinions of the experts.

**Step 2:** Comparing decision makers score. The linguistic definition to compare dimensions and CSFs is given in Table 2. If a decision maker considers first attribute to be of strong importance over the second then first gets (4, 5, 6) and the second will get (1/6, 1/5, 1/4). This results in pairwise comparison matrix as shown in (6) (where ${\tilde{a}}_{ij}^{k}$ represents rating of kth DMs for ith attribute over jth attribute). The pairwise comparison matrix is updated as in (8) using (7) that averages the ratings of k DMs.

**Table 2:** Linguistic definition and fuzzy triangular scale [95].

Intensity of importance	Linguistic Definition	Fuzzy Triangular Numbers
1	Equal importance (EI)	(1, 1, 1)
3	Weak importance of one over the other (WI)	(2, 3, 4)
5	Strong importance (SI)	(4, 5, 6)
7	Very strong importance (VSI)	(6, 7, 8)
9	Absolute importance	(9, 9, 9)
2, 4, 6, 8	Intermediate scales	(1, 2, 3), (3, 4, 5), (5, 6, 7), (7, 8, 9)

$${\tilde{A}}^{k}=\left[\begin{array}{ccc} {\tilde{a}}_{11}^{k} & \cdots & {\tilde{a}}_{1n}^{k}\\ \vdots & \ddots & \vdots \\ {\tilde{a}}_{n1}^{k} & \cdots & {\tilde{a}}_{nn}^{k} \end{array}\right]$$ (6)

$$\tilde{a_{ij}}=\frac{\sum_{k=1}^{K}{\tilde{a}}_{ij}^{k}}{K}$$ (7)

$$\tilde{A}=\left[\begin{array}{ccc} {\tilde{a}}_{11} & \cdots & {\tilde{a}}_{1n}\\ \vdots & \ddots & \vdots \\ {\tilde{a}}_{n1} & \cdots & {\tilde{a}}_{nn} \end{array}\right]$$ (8)

**Step 3:** Consistency in comparison matrix: Saaty (1980) [87], used a consistency index (CI) to measure the consistency in the verdicts of DMs in comparison matrix. Similarly, for fuzzy AHP as per article [93], CI may be estimated. Firstly, the averaged TFNs in (8) need to be converted into crisp numbers using (9) [93] and will lead to crisp comparison matrix A. The priority vector or normalized principal eigen vector W, showing normalized relative weights for criteria can be derived from A using (10). It is row averaged value of a column normalized matrix generated from A. Thereafter, matrix X, showing the weighted sum criteria can be obtained using (11). The maximum eigen value *λ*_*max*_ can be calculated from (12) that is based on Theorem *AW* = *λW* [94]. Now, CI and consistency ratio (CR) can be calculated from (13) and (14), respectively. Random consistency index (RI) for a size n can be taken from Table 3. The value of CR must be less than 0.1 in order for the acceptable consistency in the judgments of DMs [87].

**Table 3:** Random consistency index [87].

Number of Variables (n)	3	4	5	6	7	8	9
**Random Index RI (n)**	0.58	0.9	1.12	1.24	1.32	1.41	1.45

$$M_{crisp}=\frac{a+4b+c}{6}\;for\;any\;\text{TFN}\;\tilde{M}$$ (9)

$$w_{i}=\frac{\sum_{j=1}^{n}\frac{a_{ij}}{\sum_{x=1}^{n}a_{xi}}}{n}$$ (10)

X=AW (11)

$$\lambda_{max}=\frac{\sum_{i=1}^{n}\frac{x_{i}}{w_{i}}}{n}$$ (12)

$$CI=\left(\lambda_{max}-1\right)/\left(n-1\right)$$ (13)

CR=CI/RI(n) (14)

**Step 4:** Fuzzy geometric mean matrix is defined for attributes using (15). Thereafter Fuzzy weights of each attribute is calculated using (16).

$${\tilde{r}}_{i}={\left(\overset{n}{\underset{j=1}{\prod }}{\tilde{a}}_{ij}\right)}^{\frac{1}{n}},\;i=1,\;2,\;\ldots ,\;n$$ (15)

$${\tilde{w}}_{i}={\tilde{r}}_{i}\;⊗\;{\left({\tilde{r}}_{1}\;⊕\;{\tilde{r}}_{2}\;⊕\ldots \;⊕{\tilde{r}}_{n}\right)}^{-1}=\left(lw_{i},\;mw_{i},\;nw_{i}\right)$$ (16)

Here *lw*_*i*_, *mw*_*i*_, *nw*_*i*_ define the lower, middle and upper value of fuzzy weight of *w*_*i*_.

**Step 5:** De-fuzzy number for FTNs is calculated using center of area method by using (17) and normalized using (18).

$$M_{i}=\frac{lw_{i}+\;mw_{i}+\;nw_{i}}{3}$$ (17)

$$N_{i}=\frac{M_{i}}{\sum_{i=1}^{n}M_{i}}$$ (18)

### FUCOM

A relatively newer approach for multi-criteria decision making is full consistency model (FUCOM) [96] method that belongs to subjective determining of weights of criteria. It reduces the number of pairwise comparison and offers the validation through deviation from maximum consistency (DMC). It also allows for the flexibility of adoption of measurement scale as per expert preferences. It is operationalized in three steps and leads to a mathematical model to be solved by researchers preferred tools.

**Step 1:** Firstly, the set of criteria $C=\left\{C_{1},C_{2},\cdots ,C_{n}\right\}$ are ranked by decision makers (DMs) as per their expected weights from highest to lowest.

$$C_{j\left(1\right)}>C_{j\left(2\right)}>\cdots >C_{j\{k)}$$ (19)

where k represents the rank of the observed criteria

**Step 2:** Thereafter, the comparative priority $\left(\varphi_{k/\left(k+1\right)}\right)$ where k∈(1,n) represents the ranks of the criteria, is estimated. The comparative priority $\left(\varphi_{k/\left(k+1\right)}\right)$ of an evaluation criterion signifies the advantage of criterion of the *C*_*j*{*k*)_ rank in comparison to the criterion of the *C*_*j*{*k*+1)_ rank.

$$\Phi =\left(\varphi_{1/2},\varphi_{2/3},\cdots ,\varphi_{k/\left(k+1\right)}\right)$$ (20)

DMs based on their subjective preferences assign comparative priority $\left(\varphi_{k/\left(k+1\right)}\right)$ of ranked criteria.

**Step 3:** In this step the values of weights for the criteria is estimated ${\left(w_{1},w_{2},\cdots ,w_{n}\right)}^{T}$ subject to following two conditions:

- that the ratio of weights is equal to the comparative priority such as follows:
  $$\frac{w_{k}}{w_{\left(k+1\right)}}=\varphi_{k/\left(k+1\right)}$$ (21)
- and also, weights should satisfy the mathematical transitivity such as follows:

$$\frac{w_{k}}{w_{\left(k+2\right)}}=\varphi_{k/\left(k+1\right)}⊗\varphi_{\left(k+1\right)/\left(k+2\right)}$$ (22)

For the maximization of consistency, the DFC (*χ*) should be minimized which leads to the following model to be solved.

$$\begin{array}{l} \text{min}\;\chi \\ s.t.\\ \left|\frac{w_{k}}{w_{\left(k+1\right)}}-\varphi_{k/\left(k+1\right)}\right|\leq \chi ,\;\forall j\\ \left|\frac{w_{k}}{w_{\left(k+2\right)}}-\varphi_{k/\left(k+1\right)}⊗\varphi_{\left(k+1\right)/\left(k+2\right)}\right|\leq \chi ,\;\forall j\\ \sum_{j=1}^{n}w_{j}=1,\;\forall j\\ w_{j}\geq 0,\;\forall j \end{array}$$ (23)

All these steps are repeated for each criteria and sub-criteria, and for each DM. Finally, the weights calculated for each DM can be aggregated by taking their average [97].

## Application of Fuzzy AHP and FUCOM

**Fig 2:**
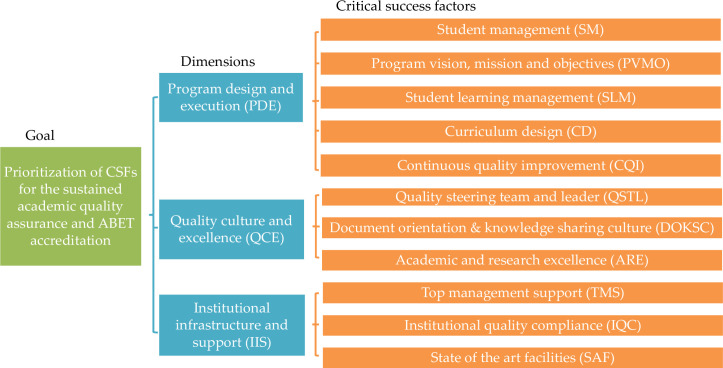
Multi-criteria decision making model for CFSs of academic quality assurance and ABET accreditation.

The goal in this research is the prioritization of CSFs for the sustained academic quality assurance quality assurance and ABET accreditation. The observation at KKU, literature review and experts’ opinions have helped develop the hierarchical structure of the defined goal. Therefore, goal at top level, dimensions of academic quality assurance and ABET accreditation are at middle level and bottom level consists of CSFs in each dimension, Fig 2. The following sections illustrate upon the implementation of Fuzzy AHP and FUCOM.

### Implementation of Fuzzy AHP

The step 1 requires to develop the hierarchical structure of goal as given in Fig 2 and described above. Proceeding to step 2, three decision makers DM_1_, DM_2_ and DM_3_ conducted pair-wise comparison for four sets of variables, first being dimensions and the rest three being their CSFs. The DMs used the linguistic definition and corresponding TFNs given in Table 2, and the comparison results are shown in Tables 4–7. Thereafter the remaining calculations represented by equation (1) to (18) in steps 2–5 were simulated in Microsoft Excel software. Tables 4–7 also contain weights obtained for dimensions and their CSFs. The consistency ratios for all four variables’ set are less than 0.1, hence shows acceptable consistency in the judgments of decision makers, Table 8. The hierarchical structure proposed in Fig 2 is not homogenous such that the number of sub-criteria at second level is not same under each dimensions and will lead to unfair global weights [98]. Therefore, the CSFs, SM and PVMO in PDE dimension, having the lowest weights of 0.0689 and 0.1171 have been dropped and the sum of their weights have been distributed equally to other three CSFs namely SLM, CD and CQI, Table 9. Finally, the global weight for each CSF is estimated by the product of its local weight and dimension weight, Table 9. These global weights define the rank of CSF, highest weight gets first rank and subsequent ranks are assigned based on decreasing order of weights, Table 9.

**Table 4:** Pair-wise comparison of dimensions of academic quality assurance and ABET accreditation and weights.

Dimensions	DMs	PDE	QCE	IIS	Weights
PDE	DM_1_	(1, 1, 1)	(1, 2, 3)	(1, 1, 1)	0.3900
	DM_2_	(1, 1, 1)	(1, 2, 3)	(1, 1, 1)	
	DM_3_	(1, 1, 1)	(1, 2, 3)	(1, 1, 1)	
QCE	DM_1_	(1/3, 1/2, 1)	(1, 1, 1)	(1/3, 1/2, 1)	0.2418
	DM_2_	(1/3, 1/2, 1)	(1, 1, 1)	(1/3, 1/2, 1)	
	DM_3_	(1/3, 1/2, 1)	(1, 1, 1)	(1, 1, 1)	
IIS	DM_1_	(1, 1, 1)	(1, 2, 3)	(1, 1, 1)	0.3682
	DM_2_	(1, 1, 1)	(1, 2, 3)	(1, 1, 1)	
	DM_3_	(1, 1, 1)	(1, 1, 1)	(1, 1, 1)	

**Table 5:** Pair-wise comparison of CSFs in Program Design and Execution (PDE) dimension and weights.

CSFs in PDE	DMs	SM	PVMO	SLM	CD	CQI	Weights
SM	DM_1_	(1, 1, 1)	(1/4, 1/3, 1/2)	(1, 1, 1)	(1/5, 1/4, 1/3)	(1/5, 1/4, 1/3)	0.0689
	DM_2_	(1, 1, 1)	(1/3, 1/2, 1)	(1/4, 1/3, 1/2)	(1/6, 1/5, 1/4)	(1/5, 1/4, 1/3)	
	DM_3_	(1, 1, 1)	(1/3, 1/2, 1)	(1/4, 1/3, 1/2)	(1/6, 1/5, 1/4)	(1/5, 1/4, 1/3)	
PVMO	DM_1_	(2, 3, 4)	(1, 1, 1)	(1/3, 1/2, 1)	(1/5, 1/4, 1/3)	(1/4, 1/3, 1/2)	0.1171
	DM_2_	(1, 2, 3)	(1, 1, 1)	(1, 2, 3)	(1/5, 1/4, 1/3)	(1/4, 1/3, 1/2)	
	DM_3_	(1, 2, 3)	(1, 1, 1)	(1/3, 1/2, 1)	(1/5, 1/4, 1/3)	(1/4, 1/3, 1/2)	
SLM	DM_1_	(1, 1, 1)	(1, 2, 3)	(1, 1, 1)	(1/4, 1/3, 1/2)	(1, 2, 3)	0.1674
	DM_2_	(2, 3, 4)	(1/3, 1/2, 1)	(1, 1, 1)	(1/4, 1/3, 1/2)	(1/3, 1/2, 1)	
	DM_3_	(2, 3, 4)	(1, 2, 3)	(1, 1, 1)	(1/4, 1/3, 1/2)	(1/3, 1/2, 1)	
CD	DM_1_	(3, 4, 5)	(3, 4, 5)	(2, 3, 4)	(1, 1, 1)	(1, 1, 1)	0.3840
	DM_2_	(4, 5, 6)	(3, 4, 5)	(2, 3, 4)	(1, 1, 1)	(1, 2, 3)	
	DM_3_	(4, 5, 6)	(3, 4, 5)	(2, 3, 4)	(1, 1, 1)	(1, 2, 3)	
CQI	DM_1_	(3, 4, 5)	(2, 3, 4)	(1/3, 1/2, 1)	(1, 1, 1)	(1, 1, 1)	0.2626
	DM_2_	(3, 4, 5)	(2, 3, 4)	(1, 2, 3)	(1/3, 1/2, 1)	(1, 1, 1)	
	DM_3_	(3, 4, 5)	(2, 3, 4)	(1, 2, 3)	(1/3, 1/2, 1)	(1, 1, 1)	

**Table 6:** Pair-wise comparison of CSFs in quality culture and excellence (QCE) dimension and weights.

CSFs in QCE	DMs	QSTL	DOKSC	ARE	Weights
QSTL	DM_1_	(1, 1, 1)	(4, 5, 6)	(2, 3, 4)	0.6265
	DM_2_	(1, 1, 1)	(4, 5, 6)	(2, 3, 4)	
	DM_3_	(1, 1, 1)	(4, 5, 6)	(2, 3, 4)	
DOKSC	DM_1_	(1/6, 1/5, 1/4)	(1, 1, 1)	(1/3, 1/2, 1)	0.1115
	DM_2_	(1/6, 1/5, 1/4)	(1, 1, 1)	(1/4, 1/3, 1/2)	
	DM_3_	(1/6, 1/5, 1/4)	(1, 1, 1)	(1/5, 1/4, 1/3)	
ARE	DM_1_	(1/4, 1/3, 1/2)	(1, 2, 3)	(1, 1, 1)	0.2620
	DM_2_	(1/4, 1/3, 1/2)	(2, 3, 4)	(1, 1, 1)	
	DM_3_	(1/4, 1/3, 1/2)	(3, 4, 5)	(1, 1, 1)	

**Table 7:** Pair-wise comparison of CSFs in Institutional Infrastructure and Support (IIS) dimension and synthesized weights.

CSFs in IIS	Decision Makers	TMS	IQC	SAF	Weights
TMS	DM_1_	(1, 1, 1)	(2, 3, 4)	(2, 3, 4)	0.6094
	DM_2_	(1, 1, 1)	(2, 3, 4)	(4, 5, 6)	
	DM_3_	(1, 1, 1)	(2, 3, 4)	(2, 3, 4)	
IQC	DM_1_	(1/4, 1/3, 1/2)	(1, 1, 1)	(1, 1, 1)	0.2147
	DM_2_	(1/4, 1/3, 1/2)	(1, 1, 1)	(1, 2, 3)	
	DM_3_	(1/4, 1/3, 1/2)	(1, 1, 1)	(1, 1, 1)	
SAF	DM_1_	(1/4, 1/3, 1/2)	(1, 1, 1)	(1, 1, 1)	0.1760
	DM_2_	(1/6, 1/5, 1/4)	(1/3, 1/2, 1)	(1, 1, 1)	
	DM_3_	(1/4, 1/3, 1/2)	(1, 1, 1)	(1, 1, 1)	

**Table 8:** Consistency ratios of different set of variables.

S. No.	Variables’ set	Number of variables (n)	Random Index (RI(n))	λ_max_	Consistency Index (CI)	Consistency Ratio (CR)
1	Dimensions	3	0.58	3.09693	0.04847	0.08356
2	CSFs for PDE	5	1.12	5.44027	0.11007	0.09827
3	CSFs for QCE	3	0.58	3.09880	0.04940	0.08517
4	CSFs for IIS	3	0.58	3.08943	0.04472	0.07710

**Table 9:** Composite weight table of CFSs of academic quality assurance and ABET accreditation obtained through Fuzzy AHP.

Dimensions of CSFs	Weights	CSFs	Local Weights	Global Weights	Overall Ranking
Program design and execution (PDE)	0.3900	Student learning management (SLM)	0.2294	0.0895	5
		Curriculum design (CD)	0.4460	0.1740	2
		Continuous quality improvement (CQI)	0.3246	0.1266	4
Quality culture and excellence (QCE)	0.2418	Quality steering team and leader (QSTL)	0.6265	0.1515	3
		Document orientation and knowledge sharing culture (DOKSC)	0.1115	0.0270	9
		Academic and research excellence (ARE)	0.2620	0.0633	8
Institutional infrastructure and support (IIS)	0.3682	Top management support (TMS)	0.6094	0.2244	1
		Institutional quality compliance (IQC)	0.2147	0.0790	6
		State of the art facilities (SAF)	0.1760	0.0648	7

### Implementation of FUCOM

The same hierarchical structure defined previously in Fig 2 has been considered in implementation of FUCOM. In the first step the criteria (dimensions) and sub-criteria (CSFs) are arranged in the expected order for their weights by the DMs, Table 10. Similarly, the priorities (Φ) for criteria and sub-criteria have been derived from the DMs’ input, Table 10. In the second step, through the priorities (Φ) of criteria and sub-criteria, comparative priorities (*φ*_*k*/(*k*+1)_) of criteria and sub-criteria is estimated. For instance, the comparative priorities (*φ*_*k*/(*k*+1)_) for dimensions and first DM may be estimated as follows: *φ*_*PDE*/*IIS*_ = 1 *and* *φ*_*IIS*/*QCE*_ = 2.

**Table 10:** Ranks and priorities of dimensions and CSFs.

Variables’ Set	DMs	Ranks (Priorities)
Dimensions	DM_1_	PDE (1) > IIS (1) > QCE (2)
	DM_2_	PDE (1) > IIS (1) > QCE (2)
	DM_3_	PDE (1) > IIS (1) > QCE (2)
CSFs for PDE	DM_1_	CD (1) > CQI (1) > PVMO (3) > SLM (4) > SM (4)
	DM_2_	CD (1) > CQI (2) > SLM (3) > PVMO (4) > SM (5)
	DM_3_	CD (1) > CQI (2) > SLM (3) > PVMO (4) > SM (5)
CSFs for QCE	DM_1_	QSTL (1) > ARE (3) > DOKSC (5)
	DM_2_	QSTL (1) > ARE (3) > DOKSC (5)
	DM_3_	QSTL (1) > ARE (3) > DOKSC (5)
CSFs for IIS	DM_1_	TMS (1) > IQC (3) > SAF (3)
	DM_2_	TMS (1) > IQC (3) > SAF (5)
	DM_3_	TMS (1) > IQC (3) > SAF (5)

In the third step weights are estimated subject to the conditions mentioned in equations (21) and (22) such that the ratio of weights should be equal to comparative priority and weights should also satisfy the mathematical transitivity. This leads to the model given below for dimensions and first DM to be solved for maximization of consistency such that minimize DFC (*χ*). Twelve such models are developed for all four set of variables such as dimensions and their CSFs, and three DMs.

$$\begin{array}{l} \text{min}\;\chi \\ s.t.\\ \left|\frac{w_{PDE}}{w_{IIS}}-1\right|\leq \chi ,\;\left|\frac{w_{IIS}}{w_{QCE}}-2\right|\leq \chi ,\\ \left|\frac{w_{PDE}}{w_{QCE}}-2\right|\leq \chi ,\\ w_{PDE}+w_{IIS}+w_{QCE}=1,\\ w_{PDE},\;w_{IIS},w_{QCE}\geq 0 \end{array}$$

These models were solved in Python language using minimize function from scipy.optimize. The resultant weights and DFC (*χ*) are shown in Tables 11–14. The weights calculated from different DMs were aggregated by simple average, Tables 11–14.

**Table 11:** Weights of dimensions of academic quality assurance and ABET accreditation, and deviation from full consistency DFC (χ).

DMs	PDE	QCE	IIS	DFC (*χ*)
DM_1_	0.4000	0.2000	0.4000	0.0000015
DM_2_	0.4000	0.2000	0.4000	0.0000015
DM_3_	0.4000	0.2000	0.4000	0.0000015
Average Weight	0.4000	0.2000	0.4000	

**Table 12:** Weights of CSFs in Program Design and Execution (PDE), and deviation from full consistency DFC (χ).

DMs	SM	PVMO	SLM	CD	CQI	DFC (*χ*)
DM_1_	0.0882	0.1176	0.0882	0.3529	0.3529	0.000187
DM_2_	0.0876	0.1095	0.1460	0.4380	0.2190	0.000011
DM_3_	0.0876	0.1095	0.1460	0.4380	0.2190	0.000011
Average Weight	0.0878	0.1122	0.1267	0.4096	0.2636	

**Table 13:** Weights of CSFs of Quality Culture and Excellence (QCE), and deviation from full consistency DFC (χ).

DMs	QSTM	DOKSC	ARE	DFC (*χ*)
DM_1_	0.6522	0.1304	0.2174	0.000009
DM_2_	0.6522	0.1304	0.2174	0.000009
DM_3_	0.6522	0.1304	0.2174	0.000009
Average Weight	0.6522	0.1304	0.2174	

**Table 14:** Weights of CSFs of Institutional Infrastructure and Support (IIS), and deviation from full consistency DFC (χ).

DMs	TMS	IQC	SAF	DFC (*χ*)
DM_1_	0.6000	0.2000	0.2000	0.000012
DM_2_	0.6522	0.2174	0.1304	0.000009
DM_3_	0.6522	0.2174	0.1304	0.000009
Average Weight	0.6348	0.2116	0.1536	

As in Fuzzy AHP, due to non-homogeneous hierarchical structure proposed in Fig 2, the CSFs, SM and PVMO in PDE dimension, having the lowest weights of 0.0878 and 0. 1122 have been dropped and the sum of their weights have been distributed equally to other three CSFs namely SLM, CD and CQI, Table 15. Finally, the global weight for each CSF is estimated by the product of its local weight and dimension weight, Table 15. These global weights define the rank of CSF, highest weight gets first rank and subsequent ranks are assigned based on decreasing order of weights, Table 15.

**Table 15:** Composite weight table of CFSs of academic quality assurance and ABET accreditation obtained through FUCOM.

Dimensions of CSFs	Weights	CSFs	Local Weights	Global Weights	Overall Ranking
Program design and execution (PDE)	0.4000	Student learning management (SLM)	0.1934	0.0774	6
		Curriculum design (CD)	0.4763	0.1905	2
		Continuous quality improvement (CQI)	0.3303	0.1321	3
Quality culture and excellence (QCE)	0.2000	Quality steering team and leader (QSTL)	0.6522	0.1304	4
		Document orientation and knowledge sharing culture (DOKSC)	0.1304	0.0261	9
		Academic and research excellence (ARE)	0.2174	0.0435	8
Institutional infrastructure and support (IIS)	0.4000	Top management support (TMS)	0.6348	0.2539	1
		Institutional quality compliance (IQC)	0.2116	0.0846	5
		State of the art facilities (SAF)	0.1760	0.0648	7

### Results and sensitivity analysis

The application of Fuzzy AHP and FUCOM, multi-criteria decision making (MCDM) methods with the help of expert opinions of decision makers have provided almost similar prioritization of dimensions and their CSFs for sustained academic quality assurance and ABET accreditation. The relative importance of the dimensions is Program design and execution (PDE) > Institutional infrastructure and support (IIS) > Quality culture and excellence (QCE) as the corresponding weights are 0.3900 > 0.3682 > 0.2418 for Fuzzy AHP, Table 9 and 0.4000 > 0.2000 > 0.4000 for FUCOM, Table 15. Therefore, the Program design and execution has the highest influence followed by Institutional infrastructure and support. And, Quality culture and excellence has relatively lower importance among other dimensions.

**Fig 3:**
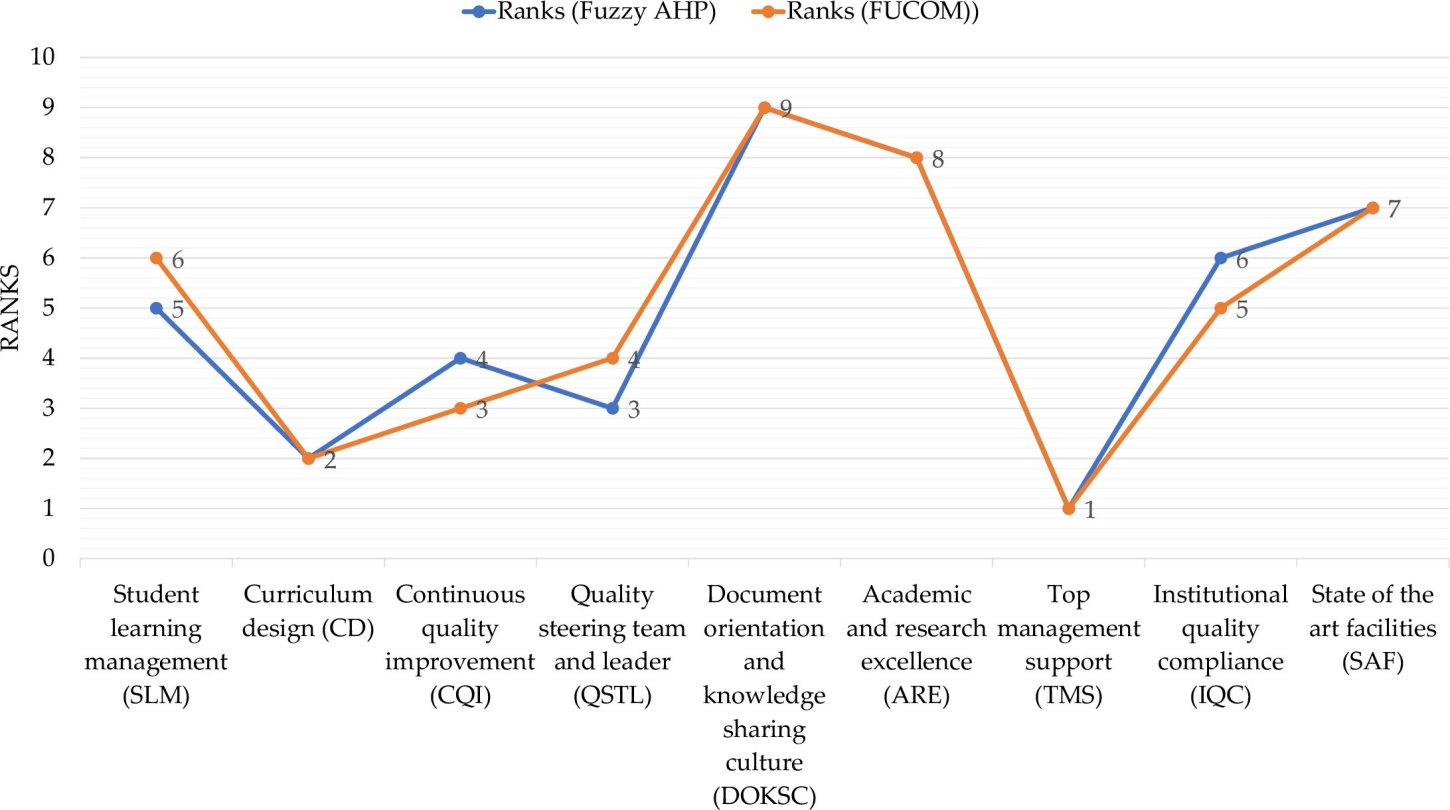
Ranks and weights of CFSs of academic quality assurance and ABET accreditation.

Similarly, the overall rankings of CSFs for both Fuzzy AHP and FUCOM have been shown in Fig 3. The top-ranking CSFs having higher influences are Top management support (TMS), Curriculum design (CD), Quality steering team and leader (QSTL), and Continuous quality improvement (CQI). The CSFs having relatively moderate influences are Institutional quality compliance (IQC) and Student learning management (SLM). The CSFs having comparatively low influences are State of the art facilities (SAF), Academic and research excellence (ARE) and Document orientation and sharing culture (DOKSC). Whereas, Program vision, mission and objectives (PVMO) and Student management (SM) dropped from ranking due to lowest weights and making the hierarchical structure homogenous, may be considered to have lowest influence. This grouping doesn’t undermine the significance of any of the CSFs rather signifies the resource requirements.

**Fig 4:**
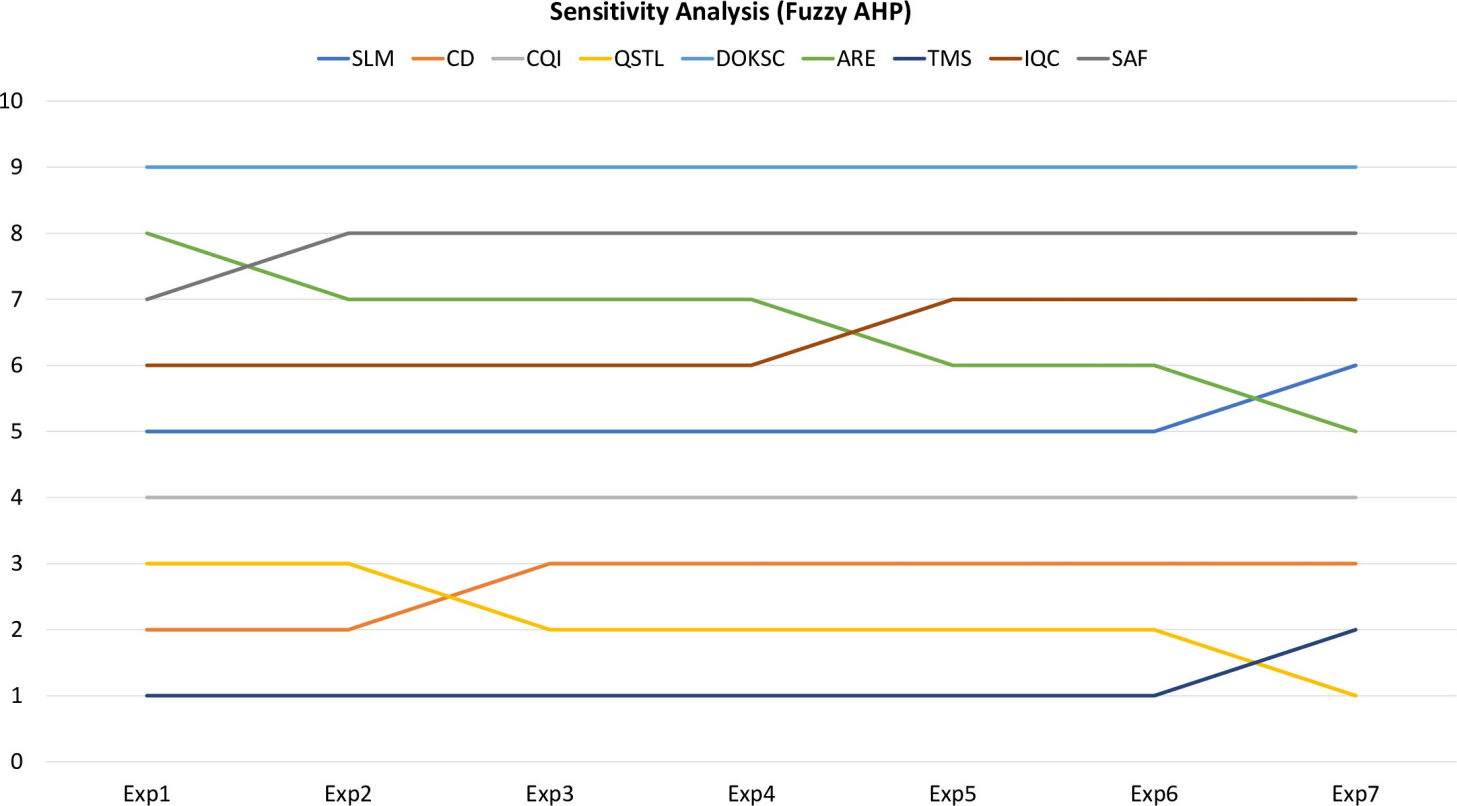
Sensitivity analysis for the ranks of CFSs for seven experiments for Fuzzy AHP.

**Fig 5:**
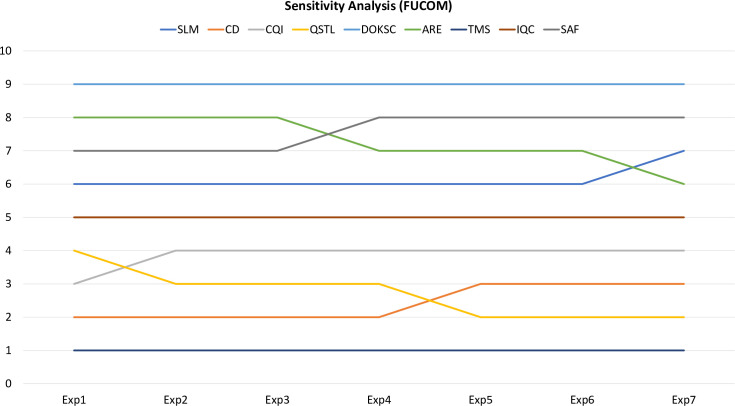
Sensitivity analysis for the ranks of CFSs for seven experiments for FUCOM.

The sensitivity analysis was performed for both Fuzzy AHP and FUCOM methods by varying the weights of dimensions of CSFs by maintaining their order such that PDE > IIS > QCE. The results are shown in Figs 4 and 5 for Fuzzy AHP and FUCOM respectively. For Fuzzy AHP, ARE shows maximum rank change of 3, followed by QSTL with rank change of two and rest of the seven CSFs show either no rank change or rank change of one for seven experiments. Similarly, for FUCOM, ARE and QSTL show maximum rank change of two, and rest of the seven CSFs show either no change or change of one rank for seven experiments. Therefore, the rankings are slightly sensitive to the changes of weights of dimensions. Further, the Spearman’s coefficient of correlation was calculated to study the correlation between rankings [97, 99]. The coefficient was calculated comparing the initial rankings with that of other experiments. For Fuzzy AHP, coefficient values range between 0.9833 and 0.85 with average value of 0.9390, hence showing extremely high correlation. Similarly, for FUCOM, coefficient values range between 0.9833 and 0.9000 with average value of 0.9500, hence showing extremely high correlation. Therefore, it can be concluded that the proposed ranking is credible.

## Discussion and conclusions

Saudi Arabia, a GCC country, has planned to diversify its economy from hydrocarbons and set ambitious future goals such as Saudi Vision 2030. Educational institutions being the primary sources of manpower are also making significant progress and receive generous funding from the government. To bring in quality in professional education such as computing and engineering, the country has set up an organization NCAAA, that has MOU with ABET to develop a national accreditation system. Currently, MoE encourages institutions to get their programs ABET-accredited. ABET being one of the oldest accrediting agencies with a global presence and constantly addressing the future needs for the professional practice, is a de facto leader for setting quality standards in STEM education. ABET, having roots of almost a century, has witnessed the evolution of engineering discipline from practice orientation to design, scientific and mathematical based discipline. ABET in early 2000s has shifted its emphasis from content-based to outcome-based education. Further, ABET is increasingly making the accreditation process less prescriptive and more objective by focusing on PEOs, SOs, and CQI.

This research has identified 11 CSFs for academic quality assurance and ABET accreditation through the observation with the ABET accreditation process of multiple programs at KKU, literature review and opinion of experts. These CSFs are categorized into Program design and execution, Quality culture and excellence, and Institutional infrastructure and support. Program design and execution consists of 5 CSFs namely: Student management, Program vision, mission and objectives, Student learning management, Curriculum design, and Continuous quality improvement. Student management deals with admission, transfer, advising, progression, internship credits, and graduation issues. PEOs the most fundamental accord to justify the program existence is handled under Program vision, mission and objects factor. Student learning management ensures the necessary knowledge, skills, and attitudes to be inculcated in the graduates to start professional practice and defined in SOs. SOs should reflect some of the following skills such as design, generic, professional, leadership, and system thinking. Curriculum design produces an OBL study plan consisting of hierarchically structured courses with well-defined CLOs having correspondence with the SOs. Some of the important considerations are backward design model; module-based, stand-alone and senior design approaches; structured and design guided experiences; live creative environments; extracurricular courses; ethics and entrepreneurship studies; and Bloom’s taxonomy. Continuous quality improvement requires assessment, review, and feedback on PEOs, KPIs, SOs, Curriculum, CLOs, and program performance from stakeholders and program constituencies in a closed-loop fashion.

The second dimension, Quality culture and excellence, contains 3 CSFs such as Quality steering team and leader, Document orientation and knowledge sharing culture, and Academic and research excellence. These factors are important to sustain the academic quality assurance efforts and taking the program at the global excellence level. A permanent Quality steering team and leader having requisite traits and motivation, is essential to work consistently. The Document orientation and knowledge sharing culture warrants electronic, structured, and reviewed document repositories. Academic excellence entails efficient synergies between program design and delivery to meet OBL. Different learning strategies such as active learning, collaborative learning, service learning, skill, project, game, team and case-based learning among others must be utilized. Similarly approaches like 4H, CPR, value-sensitive design and mini-project may also be integrated. Funded research projects may also add resources to provide learning opportunities. The third dimension, Institutional infrastructure and support category illustrates three important factors Top management support, Institutional quality compliance, and State of the art facilities. The Top management support shows a positive outlook towards maintaining high-quality standards and commitment of needed resources. organizational structure to promote quality and develop the best resources for the success of the program. Constitution of organization or university level permanent executive division can ensure standardized or Institutional quality compliance across the programs. State of the art facilities are the most visible indicator of the success of an institution and helps to sustain the excellence in programs in multiple ways.

Further, the decision makers participating in Fuzzy AHP and FUCOM, agreed with the significance of all of the CSFs. FUCOM is a recent method in subjective MCDM class that offers built-in consistency, reduced pair-wise comparisons and flexibility of measurement scale. The decision makers input in terms of pair-wise comparison of dimensions and CSFs helped in establishing their prioritization and rankings. The application of Fuzzy AHP and FUCOM has prioritized the dimensions of CSFs as Program design and execution, Institutional infrastructure and support and Quality culture and excellence in decreasing order. Similarly, the ranking of CSFs has allowed classifying CSFs into high, moderate, and low influence categories. Top management support, Curriculum design, Quality steering team and leader, and Continuous quality improvement are high influencing factors. And, Institutional quality compliance and Student learning management are categorized into moderate influencing factors. Whereas, State of the art facilities, Academic and research excellence, and Document orientation and sharing culture are grouped into comparatively low influence factors. Program vision, mission and objectives and Student management not considered in ranking have been identified to have lowest influence. This grouping doesn’t undermine the importance of any of the CSFs, rather signifies their associated resource requirements. As the Fuzzy AHP and FUCOM are based on the input of decision makers hence results may have a bias. Future research may statistically validate the results with a survey on multiple organizations. Similarly, systematic literature review may be conducted to expand the list of CSFs. Adoption of these CSFs will provide a systematic approach to sustain academic quality assurance and meet the requirements of ABET accreditation.

## Supporting information

S1 Appendix List of abbreviations. (DOCX)
