# Supplementary material for: Implementing Fuzzy AHP and FUCOM to evaluate critical success factors for sustained academic quality assurance and ABET accreditation
Source: PLoS One. 2020 Sep 17;15(9):e0239140. doi: 10.1371/journal.pone.0239140 (PMC7498038; doi:10.1371/journal.pone.0239140)
Supplement: S1 Appendix — (DOCX) [file pone.0239140.s001.docx]

**S1 Appendix. List of Abbreviations**

| ABET | Accreditation Board for Engineering and Technology |
| --- | --- |
| ADQD | Academic Development and Quality Deanship |
| AHP | Analytical hierarchy process |
| ARE | Academic and research excellence |
| ASEE | American Society for Engineering Education |
| CBL | Content-based learning |
| CD | Curriculum design |
| CEAB | Canadian Engineering Accreditation Board |
| CI | Consistency index |
| CLOs | Course learning outcomes |
| CQI | Continuous quality improvement |
| CR | Consistency ratio |
| CSFs | Critical success factors |
| DFC | Deviation from full consistency |
| DM | Decision maker |
| DOKSC | Document orientation and knowledge sharing culture |
| EC2000 | Engineering criteria 2000 |
| ECPD | Engineers’ Council for Professional Development |
| EEC | Education Evaluation Commission |
| ELD | E-Learning Deanship |
| FUCOM | Full consistency method |
| GCC | Gulf Cooperative Council |
| IIS | Institutional infrastructure and support |
| IQC | Institutional quality compliance |
| KKU | King Khalid University |
| KPIs | Key performance indicators |
| LMS | Learning management system |
| MCDM | Multi-criteria decision making |
| MoE | Ministry of Education |
| MOU | Memorandum of understanding |
| MRA | Mutual recognition agreement |
| NCAAA | National Center for Academic Accreditation and Assessment |
| OBL | Outcome-based learning |
| PDE | Program design and execution |
| PEOs | Program educational objectives |
| PVMO | Program vision, mission and objectives |
| QCE | Quality culture and excellence |
| QSTM | Quality steering team and leader |
| RI | Random consistency index |
| SAF | State of the art facilities |
| SDL | Saudi Digital Library |
| SLM | Student learning management |
| SM | Student management |
| SOs | Student outcomes |
| SSR | Self-study report |
| STEM | Science, technology, engineering and mathematics |
| TFN | Triangular fuzzy number |
| TMS | Top management support |
